# Quorum Sensing Is Required for the Colony Establishment of a Plant Phyllosphere Bacterium Rhodopseudomonas palustris Strain GJ-22

**DOI:** 10.1128/aem.00487-23

**Published:** 2023-06-05

**Authors:** Weixing Zhang, Qianze Peng, Lijie Chen, Zepei Gu, Zhuoxin Liu, Deyong Zhang, Ju’e Cheng, Limin Zheng, Ang Chen, Yong Liu, Pin Su

**Affiliations:** a State Key Laboratory of Hybrid Rice and Institute of Plant Protection, Hunan Academy of Agricultural Sciences, Changsha, China; b Long Ping Branch, Hunan University, Changsha, China; University of Tennessee at Knoxville

**Keywords:** phyllosphere, bacterial quorum sensing mechanism, cell aggregation

## Abstract

The phyllosphere presents a hostile environment for many biocontrol agents; however, it is as significant as is the rhizosphere for plant health. Deploying biocontrol bacteria into the phyllosphere can efficiently suppress diseases; however, the lack of knowledge on the phyllosphere adaptive traits of biocontrol bacteria poses challenges. In this study, we demonstrated that Rhodopseudomonas palustris GJ-22 colonizes the phyllosphere by forming cell aggregates. The formation of cell aggregates required the production of exopolysaccharides (EPS), which depended on the function of the *rpaI-rpaR* quorum sensing (QS) mechanism, mediated by the signaling molecule *p*-coumaroyl-HSL (*p*C-HSL). The mutation of the EPS biosynthesis gene *Exop1* or the signaling molecule biosynthesis gene *rpaI* compromised the ability of GJ-22 to tolerate reactive oxygen intermediates (ROIs), such as H_2_O_2_, *in vitro* and to form cell aggregates *in vivo*. Collectively, the results revealed that QS mediates EPS production and consequently leads to bacterial cell aggregation.

**IMPORTANCE** Quorum sensing is used by various bacteria for coordinating the multiplication of bacterial cells in a group and for modulating the behaviors of surrounding microbial species. Host plants can benefit from this interspecies modulation, as it can disrupt the QS circuits of pathogenic bacteria. Some N-acyl homoserine lactone- (AHL-) producing bacteria that were introduced into the phyllosphere as biocontrol agents may establish AHL-based crosstalk with indigenous microbes to steer the nutritional and microecological conditions toward their own and the host plant’s benefit. Here, we showed that biocontrol bacteria introduced into the phyllosphere require a functioning QS circuit to establish colonies and suppress pathogens. Furthermore, our findings provoked a broader investigation into the role of the QS circuit in beneficial microorganism-plant interactions.

## INTRODUCTION

The phyllosphere, mostly consisting of leaf surfaces, is the first line of defense against many plant pathogens. Moreover, it is a habitat for certain bacteria that benefit plant health and defense (1–3). Deploying biocontrol bacteria into this important niche is an effective strategy by which to obtain a complementary control spectrum to the current biocontrol approaches, which mainly consist of plant growth-promoting rhizobacteria (PGPR)-based technologies and mainly help control soilborne diseases (4). However, the phyllosphere is often regarded to be hostile to the introduced biocontrol strains, mainly due to the fluctuating physical conditions, competition with each other, limited nutrition and water, and exposure to UV radiation, reactive oxygen species (ROS), and toxins that are produced by plants and other microbes (5–8). Such unfavorable environmental conditions have dramatically reduced the survival rates and the population sizes of biocontrol strains. Therefore, the exhibition of the biocontrol functions depends on the sustainable colonization of the phyllosphere by biocontrol agents (9). Hence, colonization needs to be urgently improved for the further development of phyllosphere biocontrol strategies; however, the approaches by which to serve this purpose are scarce.

Phyllosphere bacteria usually do not exist as solitary cells or small groups, and they form aggregates at the junctions of epidermal cells, along the veins, and at the bases of trichomes (10). Importantly, bacterial cells within the aggregate can coordinate the expression of some traits in a cell density-dependent manner that is known as quorum sensing (QS) (11). QS is a mechanism by which bacteria use small, diffusible molecules to monitor their local population density and alter gene expression at high population densities (12). The LuxI-LuxR-type QS system plays a crucial role in the epiphytic fitness of many plant pathogens and symbionts by regulating the traits required for epiphytic colonization (13). The LuxI family protein synthesizes the QS signaling molecule, namely, *N*-acyl homoserine lactone (AHL), which is also known as an autoinducer (AI). *N*-acyl homoserine lactone diffuses into the bacterial habitat and concomitantly accumulates along with the growing bacterial population (14). The transcription factors of the LuxR family detect the accumulated AHLs; a conformational change occurs in the LuxR protein upon binding with AHL, and the probability of their interaction is determined by the relative concentrations of the two components. This conformational change allows the LuxR protein to activate various QS-targeted gene expressions, including the gene *luxI*. Extracellular polymeric substance (EPS) acts as a molecular glue and allows for adhesion both among cells and between cells and plant surfaces, thereby facilitating the formation of aggregates. This cell aggregate structure protects bacterial cells from a wide range of environmental stresses. Therefore, manipulating EPS production by tampering with bacterial QS regulation significantly affects bacterial epiphytic fitness. For instance, the mutation of the AHL synthase gene *ahlI* or the transcription factors gene *ahlR* in Pseudomonas syringae impairs EPS production, thereby resulting in increased cell susceptibility to reactive oxygen intermediates (ROIs) (15). The formation of aggregates is considered to be an overarching strategy for the survival of bacteria under biotic and abiotic stresses in the phyllosphere. Hence, biocontrol strains may also use this strategy in the phyllosphere to establish colonies and exert biocontrol functions.

Rhodopseudomonas palustris is a purple nonsulfur photosynthetic bacterium belonging to the class Alphaproteobacteria. R. palustris strains have gained enormous attention from scientists due to their extraordinary metabolic versatility (16). They are capable of nitrogen fixation, hydrogen gas production, and the degradation of aromatic compounds and chlorinated pollutants, which have been intensively studied (17, 18). In our previous study, we reported a photosynthetic bacterial strain, namely, R. palustris GJ-22. Its QS signaling molecule *p*C-HSL causes induced plant systemic resistance (ISR) against plant viruses (19–21). Further research revealed that the colony establishment of GJ-22 in the phyllosphere is comprised of four stages, based on the observable morphological changes in colonies (21). Bacterial cell aggregate formation has important implications for stable colonization and the resulting exhibition of functional traits. Based on the known adaptive traits of the phyllosphere bacteria, the following hypothetical mechanism was outlined: the accumulated cells in stage II trigger the QS regulation, thereby resulting in the upregulation of EPS production, which, in turn, leads to the aggregate formation (21). To test this hypothesis, we identified a LuxIR-type QS system involving the *rpaI-rpaR* gene pair in the GJ-22 genome and proved that *rpaI-rpaR* genes were required for the upregulation of EPS production and cell aggregate formation (21). This study provides valuable information for the future development of methods aiming at improving the colonization and performance of phyllosphere biocontrol agents.

## RESULTS

### Identification of a LuxIR-type QS system in the R. palustris GJ-22 genome.

The R. palustris GJ-22 genome possesses a *luxI-luxR* gene pair (locus tag: GJ-22_21460 and 21465) that is homologous to the *rpaI* (*rpa* 0320) and *rpaR* (*rpa* 0321) in the R. palustris CGA009 genome. The *luxI* type gene in CGA009, *rpaI*, encodes *p*C-HSL synthase, which synthesizes the QS signaling molecule (*p*C-HSL). The LuxR type gene *rpaR* encodes a *p*C-HSL cognate receptor protein that regulates the transcription of *lux* structural genes upon binding to *p*C-HSL. The homologs of RpaI and RpaR proteins (here called RpaI and RpaR) of GJ-22 share 94% and 93.8% similarity with the RpaI and RpaR proteins of CGA009, respectively (Tables S1–S3 in Supplementary File 1). In the GJ-22 genome, the *rpaI* and *rpaR* are located adjacently and transcribe in the same orientation. There is an 86-bp sequence between the *rpaR* translational stop codon and the *rpaI* transcriptional start site. In this sequence, two *lux* box-like inverted repeat elements are present at −76 and −35 bp upstream of the *rpaI* start codon (ATG). This *rpaR-lux box-rpaI* gene arrangement in GJ-22 was identical to that in CGA009 (Fig. S3 in Supplementary File 1). R. palustris uses an exogenous plant-derived substrate, namely, *p*-coumarate, to synthesize *p*C-HSL. The *p*C-HSL synthase catalyzes the formation of an amide bond between S-adenosyl-l-methionine (SAM) and the side chain donor, namely, *p*-coumaroyl-CoA, to produce *p*C-HSL. In R. palustris and in many other aromatic compound-degrading bacteria, *p*-coumaroyl-CoA formation is catalyzed by *p*-coumarate-CoA ligase (*CouB*), and it is also the initial step for *p*-coumarate degradation. In the GJ-22 genome, a protein encoded by a *couB* gene homolog (locus tag: GJ-22_17460) showed >98% sequence similarity (data not shown) with other two CouB proteins (UniProtKB: Q6N1I4 and A0A2R4GZU1) that are found in R. palustris strains.

**FIG 1:**
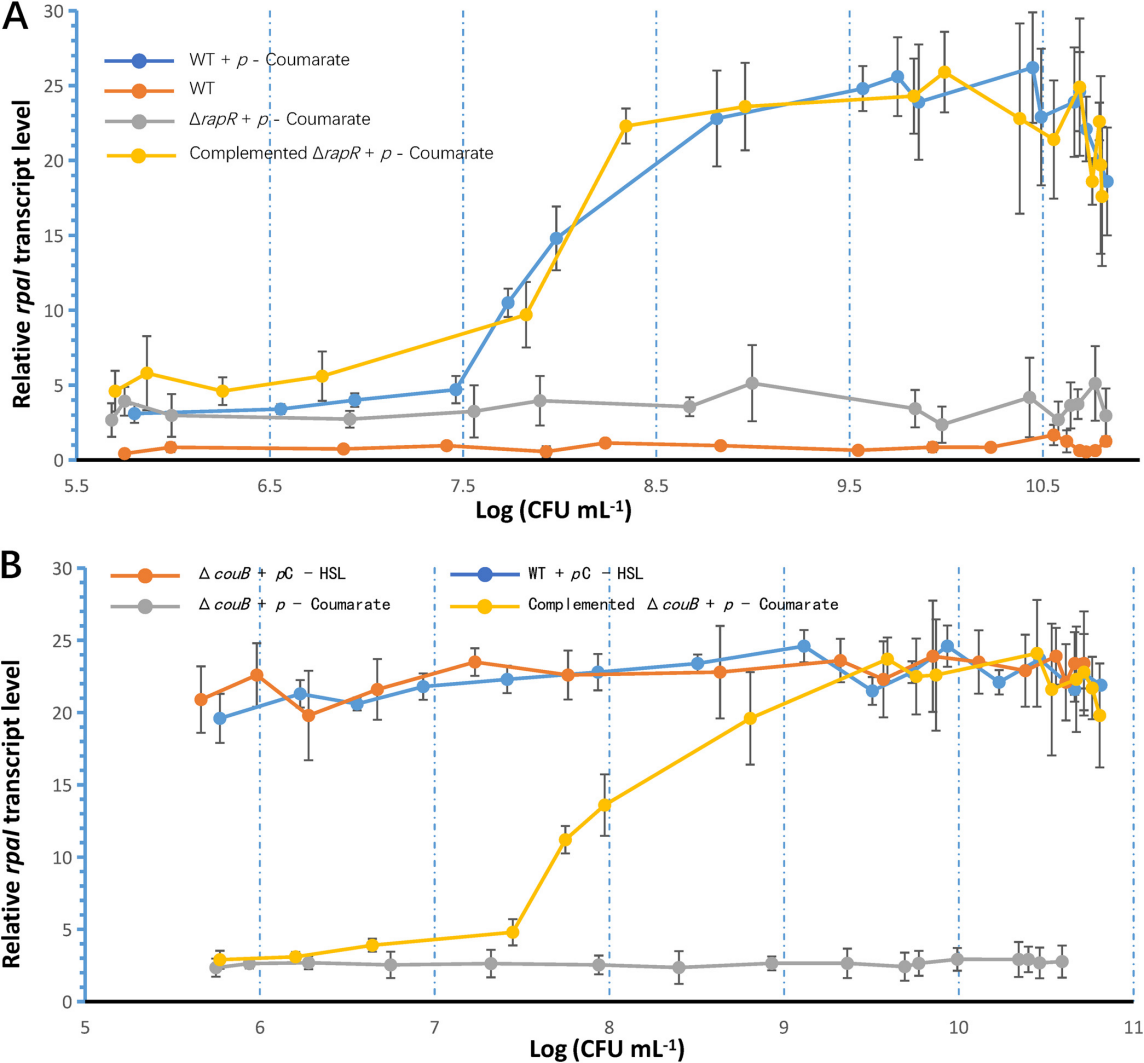
The *rpaI-rpaR* gene pair functions in a QS regulating pattern in R. palustris strain GJ-22. (A) The *rpaR* gene and *p*-coumarate are required for the cell density-dependent regulation of *rpaI* transcription. (B) *rpaI-rpaR* gene pair-regulated QS relies on *p*C-HSL synthesis. *p*-Coumarate was added into photosynthetic liquid medium at the concentration of 0.5 mM, and *p*C-HSL was added into photosynthetic liquid medium at the concentration of 250 nM. The *rapI* gene transcript level was normalized using the control transcript of the gene *rpoD.* Samples were taken every 12 h from 2 to 9 dpi. The cell density of each sampling time point was expressed as the Log(CFU mL^−1^). In this figure, WT was the wild-type strain GJ-22. Δ*rpaR* was the WT mutated by replacing *rpaR* CDS with a cassette containing the Hyg^r^ gene that followed a Lac promoter. Δ*rpaR* was complemented with the plasmid pBBR1MCS-2R. Δ*couB* was the WT mutated by replacing *couB* (a *p*-coumarate-CoA ligase, to produce a donor of synthesis *p*C-HSL) CDS with the cassette containing the Amp^r^ gene that followed a PAMP promoter. Δ*couB* was complemented with the plasmid pBBR1MCS-2couB. The data are presented as the means ± SD (shown as error bars; *n* = 5).

In a typical LuxIR-type QS system, when the concentration of the signaling molecule, namely, AHL, reaches a certain threshold due to the increasing cell density, LuxR forms a stable complex with the AI molecule, thereby resulting in the activation of *luxI* transcription. The *luxI* transcription is upregulated due to this cell density-dependent positive feedback mechanism. When GJ-22 wild-type cells were cultured in a medium that was supplemented with *p*-coumarate, the *rpaI* transcription levels increased as the cell density became greater than 10^7^ CFU mL^−1^ (Fig. 1A).

Conversely, *rpaI* transcription remained at low and constitutive levels at all cell densities when *p*-coumarate was removed from the culture medium. Similarly, *rpaI* transcription was also low in the mutant Δ*rpaR* when cultured in a medium containing *p*-coumarate. The upregulation of *rpaI* transcription was recovered as the cell density reached the threshold value when Δ*rpaR* was complemented with the plasmid pBBR1MCS-2R. To further verify the role of *p*C-HSL as an AI in GJ-22, the *couB* homolog was mutated, thereby resulting in the impaired synthesis of *p*C-HSL due to the lack of substrate *p*-coumaroyl-CoA, to observe the aberration of *rpaI* transcription (Fig. 1B). As expected, the *rpaI* transcription remained at a low level, regardless of the cell density of Δ*couB* when cultured in a *p*-coumarate-supplemented medium. The complementation of Δ*couB* with the plasmid pBBR1MCS-2couB restored the cell density-dependent upregulation of *rpaI* transcription. When the culture medium was supplemented with *p*C-HSL to simulate QS signaling at a high cell density, both the wild-type and Δ*couB* showed high levels of *rpaI* transcription at all cell density levels. Combining the known information on the *rpaI-rpaR* QS system in R. palustris, the results confirmed that the *rpaI-rpaR* gene pair in GJ-22 regulated the QS pattern when *p*C-HSL synthesis was enabled. In addition, the RpaR required *p*C-HSL to function as a transcriptional regulator.

### The *rpaI-rpaR* QS system positively regulated EPS production in R. palustris GJ-22.

**FIG 2:**
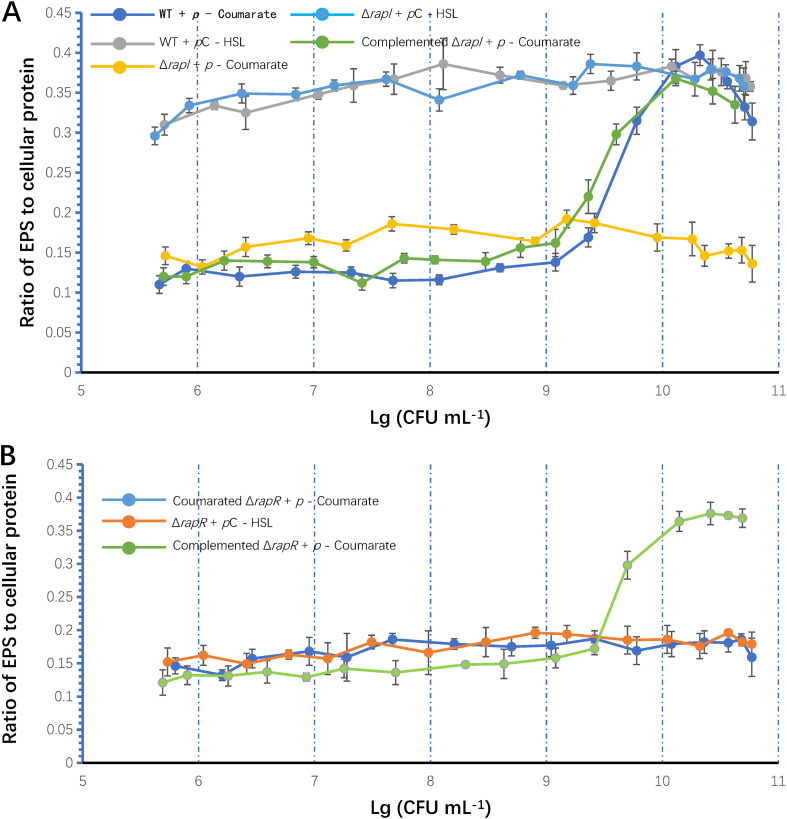
The production of EPS is regulated by the QS system in R. palustris strain GJ-22. (A) The cell density-dependent regulation of EPS production requires *p*C-HSL synthesis. (B) EPS synthesis regulation requires *rpaR*. *p*-Coumarate was added into photosynthetic liquid medium at the concentration of 0.5 mM, and *p*C-HSL was added into photosynthetic liquid medium at the concentration of 250 nM. EPS production was conveyed as the ratio of EPS to cellular protein. Samples were taken every 12 h from 2 to 9 dpi. The cell density of each sampling time point was expressed as the Log(CFU mL^−1^). WT was the wild-type strain GJ-22. The strains of mutations and complemented mutations are the same as those presented in Fig. 1. The data are presented as the means ± SD (shown as error bars; *n* = 5).

In many bacteria, the LuxIR-type QS regulates the expression of various factors, such as EPS production, which helps tolerate stress. To determine whether EPS production is regulated by *rpaI-rpaR* QS in GJ-22, the ratio of EPS to cellular protein was monitored when the wild-type cells were cultured in a medium supplemented with *p*-coumarate. The increased ratio indicated the induction of EPS production and was dependent on the cell density (Fig. 2A).

At a cell density of 10^9^ CFU mL^−1^, the wild-type showed upregulated EPS production. To prove that the regulation depends on *p*C-HSL, *rpaI* was mutated to inhibit *p*C-HSL synthesis, and Δ*rpaI* was cultured with *p*-coumarate. The inhibition of *p*C-HSL synthesis resulted in less EPS production at all cell densities. A similar pattern was observed when the wild-type was grown in a medium without *p*-coumarate (data not shown). The cell density-dependent upregulation was restored via the complementation of Δ*rpaI* with the plasmid pBBR1MCS-2I that contained the *rpaI* coding sequence and the 86-bp upstream sequence. The EPS production remained high, independent of the cell density, in both the wild-type and Δ*rpaI* when *p*C-HSL was added to the culture media to simulate QS signaling at a high cell density. In addition, Δ*rpaR* did not show such regulation under both culture conditions, with *p*-coumarate or *p*C-HSL, whereas complemented Δ*rpaR* could regulate under both conditions (Fig. 2B). These results suggest that EPS production is regulated by the *rpaI-rpaR* QS system under conditions supporting *p*C-HSL synthesis.

**FIG 3:**
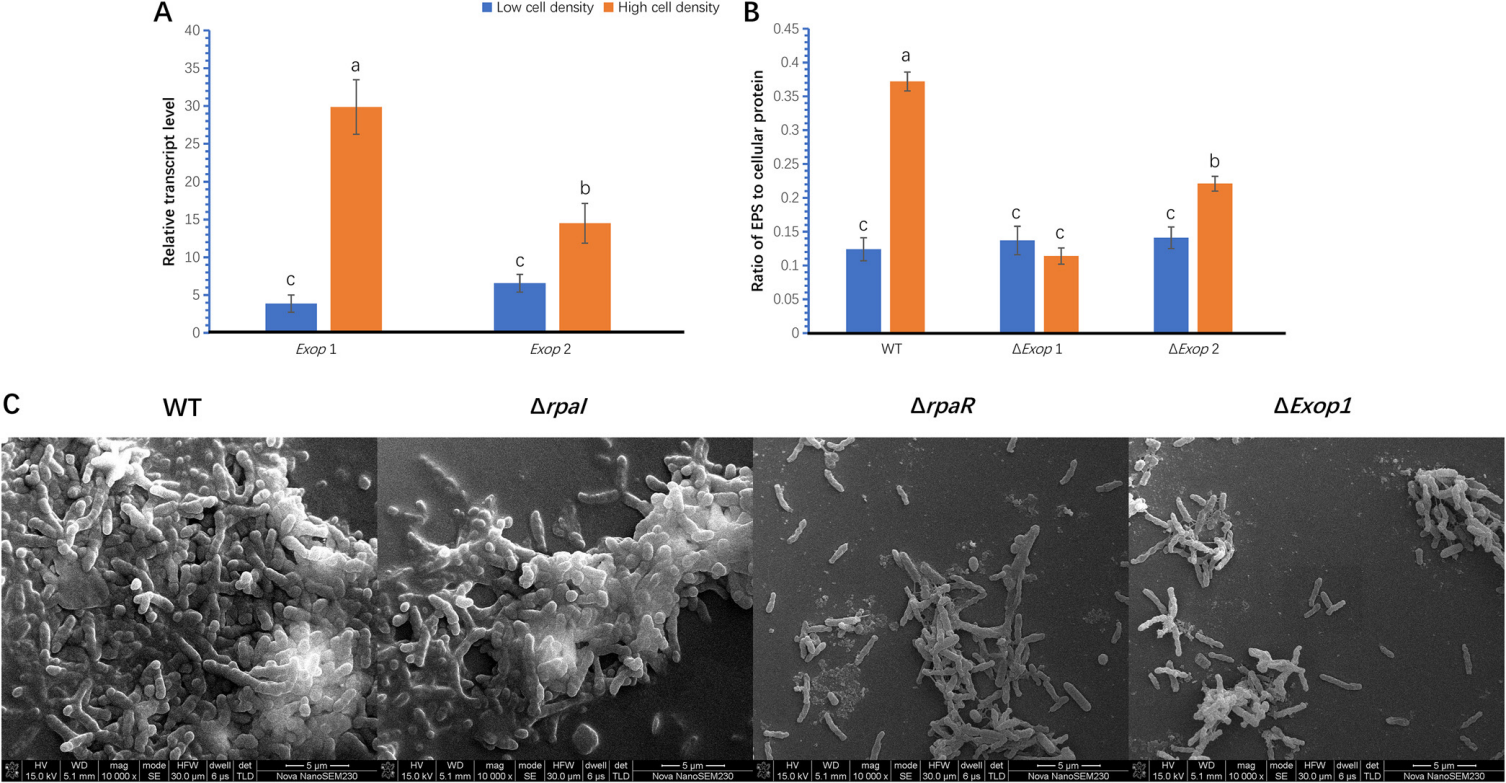
Cell aggregation required both *rpaI-rpaR* QS regulation and EPS. (A) The transcript levels of two polysaccharide export protein genes, namely, *Exop1* and *Exop2*, under low cell density and high cell density growth stages. The transcript levels were normalized by using the control transcript of the gene *rpoD.* (B) The detectable EPS production in the wild-type and in the mutant strains Δ*Exop1* and Δ*Exop2* under low cell density and high cell density growth stage. Low cell density was determined as 8.7 × 10^6^ CFU mL^−1^, and high cell density was determined as 2.6 × 10^10^ CFU mL^−1^. EPS production was conveyed as the ratio of EPS to cellular protein. Different small letters indicate statistical differences between the treatments in each group via Fisher’s LSD (*P* < 0.05). The data are presented as the means ± SD (shown as error bars; *n* = 5). (C) Scanning electron micrograph of the wild-type GJ-22 and its mutants. After 6 dpi, treatment cells were taken from a liquid culture that was supplemented with *p*C-HSL (250 nM). The scale bars represent 5 µm. The strains of the mutants are the same as those presented in Fig. 1. Δ*Exop1* was the WT mutated by replacing *Exop1* CDS with the cassette containing Hyg^r^ gene that followed a CaMV 35S promoter. Δ*Exop2* was the WT mutated by replacing *Exop2* CDS with the cassette containing the Hyg^r^ gene that followed a CaMV 35S promoter.

The transcript levels of two polysaccharide export protein genes (*Exop1* and *Exop2*) (21) were compared in cells at low (8.7 × 10^6^ CFU mL^−1^) and high (2.6 × 10^10^ CFU mL^−1^) density levels. Cells were sampled from these two growth stages to show the transcript levels both before and after the upregulation of EPS production. The transcript levels of *Exop1* and *Exop2* increased 7.71-fold and 2.2-fold, respectively, under the high cell density (Fig. 3A).

The mutation of both the genes resulted in impaired EPS production, compared with the wild-type at high cell density (Fig. 3A). However, in Δ*Exop1*, the upregulation of EPS production was completely terminated at the high cell density. Conversely, the wild-type and Δ*Exop2* showed increased EPS production by 3-fold and 1.6-fold, respectively. These results indicate that GJ-22 mainly relied on the *Exop1* protein to export polysaccharides to the outer membrane under high EPS production.

When wild-type, Δ*rpaI*, Δ*rpaR*, and Δ*Exop1* were cultured in a liquid medium containing *p*C-HSL, all strains exhibited similar growth rates (data not shown); however, a scanning electron microscopy (SEM) analysis revealed that their cell morphology varied conspicuously. At 6 dpi, wild-type and Δ*rpaI* cells were observed to be wrapped in a thick exopolymeric matrix and forming large cell clusters (Fig. 3B). Such morphology was observed neither in Δ*Exop1*, which showed impaired polysaccharide export nor in Δ*rpaR*, which was irresponsive to *p*C-HSL. The cells of both strains were loosely attached by a lesser amount of extracellular substance. This morphological difference may be due to the upregulation of EPS production in wild-type and Δ*rpaI* cells, which showed the activation of QS regulation, in contrast to the Δ*Exop1* and Δ*rpaR* cells, which showed impaired polysaccharide export or disabled QS regulation.

### The *rpaI-rpaR* QS-mediated EPS production enhanced cell tolerance to oxidative stress.

**FIG 4:**
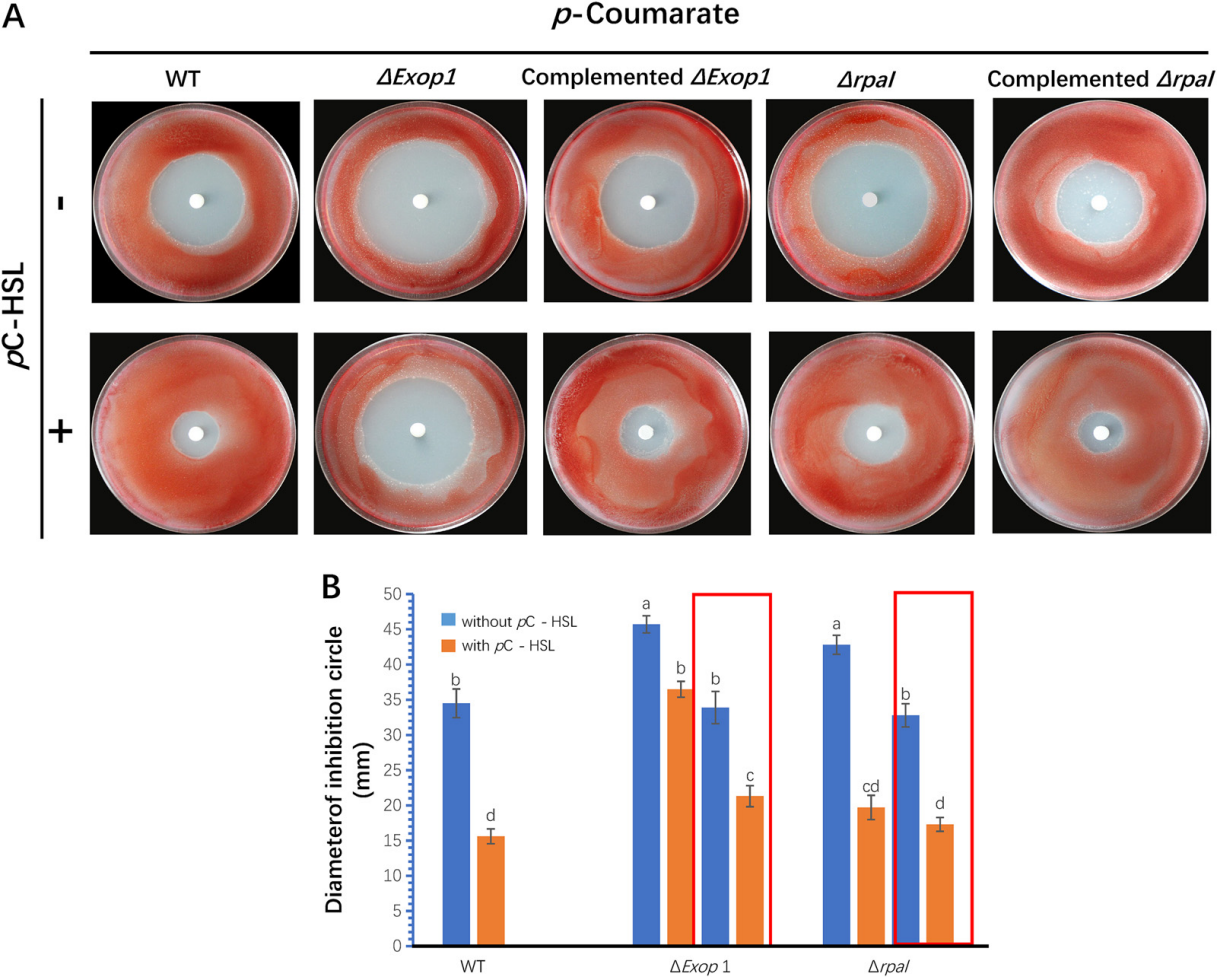
The tolerance to RIOs required both *rpaI-rpaR* QS regulation and EPS. (A) The inhibition circle of wild-type strain GJ-22 and its mutants. The strains were inoculated via pouring into the PM agar at a concentration of 6 × 10^7^ CFU mL^−1^. The filter discs placed in the plate centers were soaked with 10% (vol/vol) hydrogen peroxide/water. Photos were taken at 6 dpi. (B) Analysis of the diameters of the growth inhibition circles. The diameters were measured at 6 dpi. The columns in red boxes were values from complemented mutants that grew on the indicated media. *p*-Coumarate and *p*C-HSL were added into photosynthetic liquid medium at a concentration of 0.5 mM and 250 nM, respectively. The strains of the mutations are the same as those presented in Fig. 3. Δ*Exop1* was complemented with the plasmid pSUP202E1. Different small letters indicate statistical differences between the treatments in each group via the Fisher’s LSD (*P* < 0.05). The data are presented as the means ± SD (shown as error bars; *n* = 5).

A LuxIR-type QS system was identified in the R. palustris GJ-22 genome (Table S1–S3 in Supplementary File 1) and could positively regulate EPS production (Supplementary File 1). To investigate whether the regulation of EPS production by the *rpaI-rpaR* QS system played a role in oxidative stress tolerance, we used Δ*Exop1* (as the polysaccharide export protein mutant), the wild-type, and Δ*rpaI* for the oxidative stress tolerance assay. The susceptibility of strains to hydrogen peroxide was tested using culture mediums supplemented with or without *p*C-HSL. Hydrogen peroxide generates ROIs to inhibit bacterial growth. In a culture medium without *p*C-HSL on medium plates, Δ*Exop1* and Δ*rpaI* exhibited the greatest susceptibility to ROIs (Fig. 4A), as evidenced by the 32% and 24% larger diameters of their inhibition zones, respectively (Fig. 4B), compared with that of the wild-type. The complementation of both mutants resulted in no significant differences in the inhibition diameter (Fig. 4B) (Fisher’s LSD test, *P* > 0.05), compared with that of the wild-type. When the culture medium was supplemented with *p*C-HSL to activate QS regulation, all of the strains except Δ*Exop1* showed significantly decreased inhibition diameters, compared with the strains that were cultured without *p*C-HSL. These results suggest that *rpaI-rpaR* QS in GJ-22 enhanced the oxidative stress tolerance of the cells through the upregulation of EPS production.

### Regulation of EPS production by the *rpaI-rpaR* QS system was essential for the epiphytic fitness of R. palustris GJ-22.

**FIG 5:**
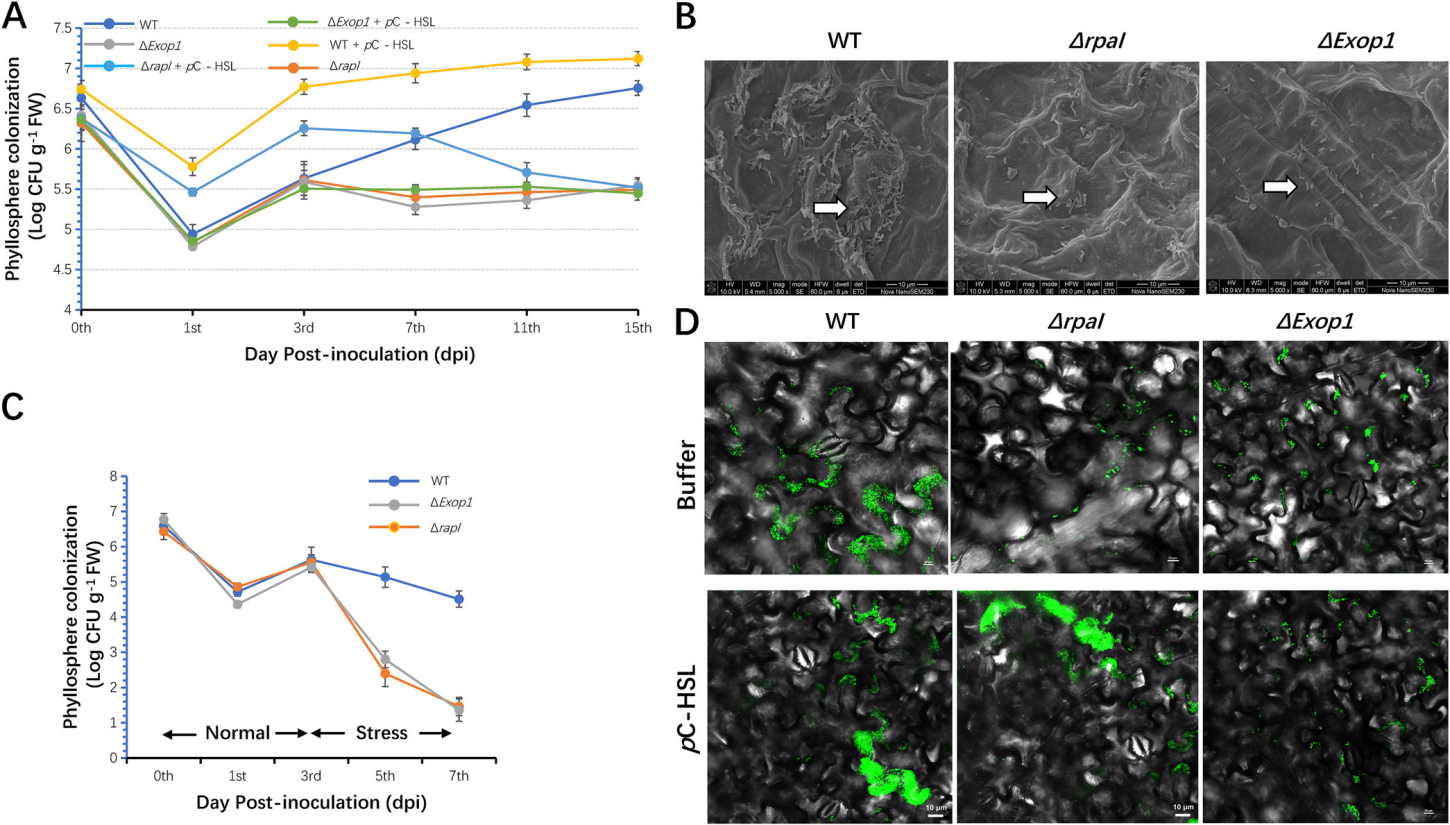
The bacteria phyllosphere colonization required the *rpaI-rpaR* QS-regulated EPS production. (A) The colonization dynamics of the GJ-22 wild-type, Δ*Exop1*, and Δ*rpaI* in the tobacco phyllosphere. Tobacco seedlings were cultivated under axenic conditions, and *p*C-HSL was added into the bacterial inoculums at a concentration of 500 nM, prior to inoculation, when indicated. (B) SEM analysis of the GJ-22 wild-type, Δ*Exop1*, and Δ*rpaI* colony morphology in the tobacco phyllosphere. Images were taken at 7 dpi. The scale bars represent 10 µm. (C) The stress tolerance varied among the GJ-22 wild-type and its mutants. Previous studies have shown that changes in temperature or humidity have an effect on QS (26). The strains were inoculated in tobacco seedlings under normal conditions, which were set as relative humidity = 85% and temperature = 28°C. The plants were transferred to stress conditions (set as relative humidity = 40%, temperature = 39°C) at 3 dpi. (D) CLSM analysis of the GJ-22 wild-type and its mutants in the tobacco phyllosphere. The wild-type, Δ*Exop1*, and ΔrpaI were labeled with eGFP via transformation with plasmid pBBR1MCS-2-pAMP-EGFP. Labeled strains were inoculated on tobacco seedlings. Inoculated leaves were sprayed with a *p*C-HSL solution at a concentration of 500 nM or with buffer at 3 dpi. Images were taken 1 day after the spray. The scale bars represent 10 µm. In this figure, the data are presented as the means ± SD (shown as error bars; *n* = 5). The strains of the mutations are the same as those presented in Fig. 3.

To determine whether the *rpaI-rpaR* QS system and its regulation of EPS production are required for the epiphytic fitness of GJ-22, we inoculated the leaf surfaces of tobacco seedlings with the wild-type, Δ*Exop1*, and Δ*rpaI*, and we observed their colonization dynamics (Fig. 5A).

From 0 to 3 dpi, the wild-type, Δ*Exop1*, and Δ*rpaI* exhibited similar colonization dynamics. The recovery of the populations of all of the strains was initiated at 1 dpi, and the population density increased until 3 dpi. A difference in density was observed after 3 dpi; the density of the wild-type proceeded to increase further, whereas those of Δ*Exop1* and Δ*rpaI* did not increase. At 15 dpi, the wild-type exhibited more than 100-fold greater density than did Δ*Exop1* and Δ*rpaI*. At 3 dpi, an SEM analysis revealed that the wild-type had formed cell aggregates in plant epidermal cell junctions, whereas Δ*Exop1* and Δ*rpaI* did not show such colony morphology. Both of the mutants stayed in the form of cell arrays in the phyllosphere (Fig. 5B). Previous studies have shown that changes in temperature or humidity affect QS (22). The inoculated plants were transferred from normal growth conditions to stress conditions at 3 dpi, thereby resulting in a significant decrease in the density of Δ*Exop1* and Δ*rpaI* in the phyllosphere; conversely, the wild-type maintained its population size (Fig. 5C). A CLSM analysis of eGFP-tagged strains revealed that the application of *p*C-HSL to plant leaves that had been inoculated with these strains at 3 dpi helped expand the colonies of Δ*rpaI*, whereas colony expansion was not observed for Δ*Exop1*, which had impaired polysaccharide export (Fig. 5D). These results suggest that the transformation of colony morphology from stage II (cell arrays) to stage III (cell aggregates) (21) was dependent on the regulation of EPS production by *rpaI-rpaR* QS and that this morphology transformation was crucial for enhancing stress tolerance and colony expansion.

**FIG 6:**
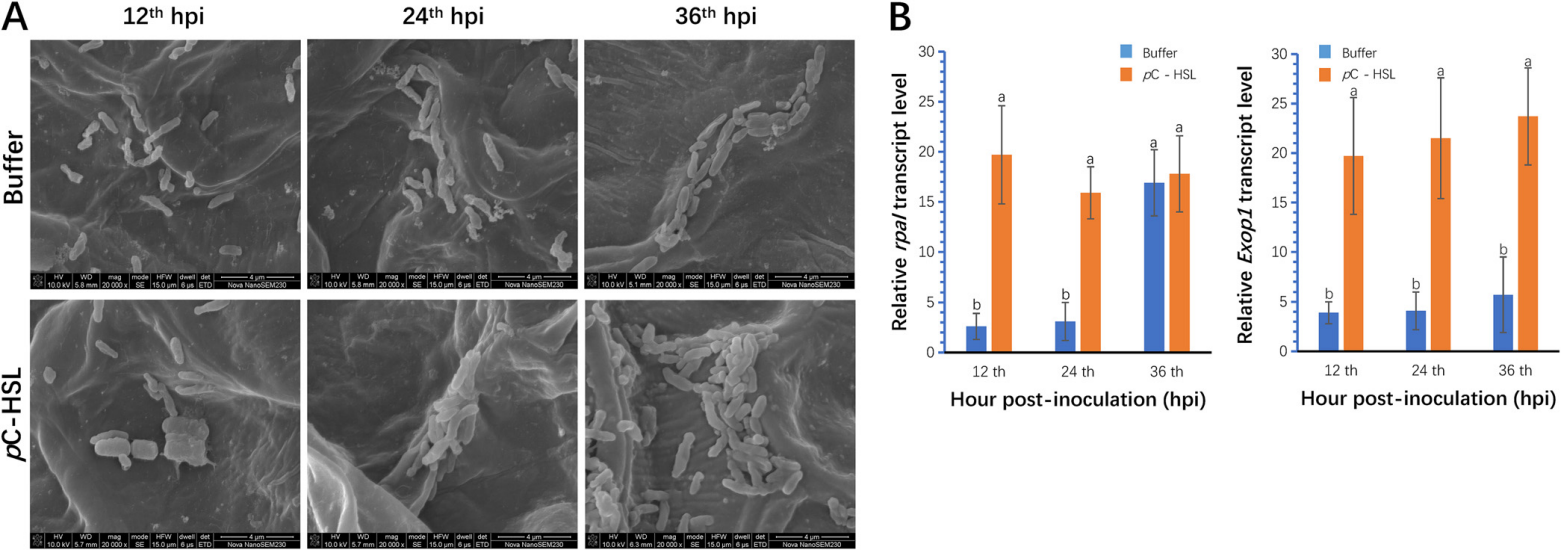
The activation of *rpaI-rpaR* QS regulation promoted cell aggregate formation in the plant phyllosphere. (A) The colony morphology difference between GJ-22 cells treated with or without *p*C-HSL in the tobacco phyllosphere. *p*C-HSL was added into the GJ-22 inoculum at a concentration of 500 nM before inoculation. The SEM analysis was carried out at 12, 24, or 36 hpi. The scale bars represent 4 µm. (B) The transcript levels of *rpaI* and *Exop1* genes at different times. Bacterial cells were collected from inoculated leaves at 12, 24, or 36 hpi for RT-PCR, using the gene *rpoD* as the reference. Different small letters indicate statistical differences between the treatments in each group via Fisher’s LSD (*P* < 0.05). The data are presented as the means ± SD (shown as error bars; *n* = 5).

To investigate whether the cell aggregate formation can be accelerated by the early activation of the *rpaI-rpaR* QS regulation mechanism, *p*C-HSL was added to the GJ-22 inoculum, and its colony morphology was analyzed using SEM (Fig. 6A).

The cells showed increased regulation of EPS production at 12 hpi, and cell aggregates formed at 36 hpi, whereas cells normally enter this stage at 60 hpi (21). The early cell aggregate formation induced by *p*C-HSL was verified by the upregulation of the *rpaI* and *Exop1* transcript levels (Fig. 6B). Without *p*C-HSL supplementation, the upregulation of the transcript levels of *rpaI* did not initiate until 36 hpi, the and *Exop1* transcript levels remained low during this period. In contrast, the addition of *p*C-HSL resulted in high transcript levels of *rpaI* and *Exop1* in GJ-22. This early cell aggregate formation in the phyllosphere was responsible for the increased population density of the wild-type and Δ*rpaI* when the inocula were supplemented with *p*C-HSL (Fig. 5A), as both of the strains could upregulate EPS production in the presence of *p*C-HSL.

## DISCUSSION

QS is a vital aspect of microbiological activity. Since the discovery of the AHL-based QS system in Vibrio fischeri (23), a whole inventory of QS-related genes and phenotypes has been unraveled in various microorganisms. QS has been demonstrated to control various adaptive traits, such as EPS production (21), plasmid transfer (24), root nodulation efficiency (25), nitrogen-fixation efficiency (26), motility (27), and virulence factors (28), in many plant-associated microorganisms. In a culture supplemented with *p*-coumarate, the transcript levels of the *rpaI* gene in GJ-22 displayed typical cell-density-dependent expression. The mutation of the *couB* gene (Supplementary File 1) inhibited regulation by the *rpaI-rpaR* QS system, thereby indicating that RpaI uses *p*-coumaroyl-CoA as an aryl donor in GJ-22. This is in concordance with the opinion that RpaI in R. palustris, BjaI in Bradyrhizobium japonicum, and BraI in stem-nodulating photosynthetic *Bradyrhizobia* may have evolved into a subfamily of *luxI* homologs that use only acyl-CoAs or aryl-CoAs for AHL synthesis (29, 30). Many symbiotic and pathogenic bacteria rely on QS-mediated EPS production to achieve epiphytic fitness. The phytopathogen Pseudomonas syringae B728a uses a 3-oxo-hexanol-homoserine lactone-based AhlI-AhlR QS mechanism to promote alginate production, which enhances bacterial oxidative stress tolerance and plant tissue maceration (31). Sinorhizobium meliloti uses the *SinI-SinR* QS mechanism to regulate EPS II synthesis, which is required for nodulation in alfalfa (32). Both the negative and positive regulation of EPS synthesis by QS in bacteria facilitates the establishment of relationships with their hosts. In this study, EPS production was positively regulated by the *rpaI-rpaR* QS mechanism in R. palustris GJ-22. This observation was in contrast to the negatively regulated EPS production by the 7,8-cis-N-(tetradecenoyl) homoserine lactone-based *CerI-CerR* QS mechanism in Rhodobacter sphaeroides, which is another purple nonsulfur photosynthetic bacterium that belongs to Alphaproteobacteria (33). It has been reported that R. palustris strains diverged into physiologically and genetically distinct ecotypes to adapt to various environmental conditions. Each ecotype possesses specific physical and chemical characteristics to adapt to different habitats. In this study, the regulation of EPS production by the *rpaI-rpaR* QS system in R. palustris GJ-22 enhanced its survival and colonization on the plant surface, similar to many other AHL-based QS systems that contribute to the epiphytic fitness of plant-associated bacteria. The results of this study suggest that the *rpaI-rpaR* QS mechanism is likely to be fine-tuned in different ecotypes of R. palustris strains to adapt to different environmental conditions. The same set of genes serves different purposes in different ecotypes of R. palustris strains or in the same strain under different environmental conditions, which highlights the metabolic complexity of this bacterium, in addition to it possessing multiple modes of metabolism.

R. palustris is nonpathogenic to plants and is mostly a free-living bacterium in soils and aquatic sediments (34, 35). The physiological and genetic insights about this bacterium are mainly focused on its lifestyle. R. palustris strains impart several beneficial effects upon plant growth through the production of phytohormones and through boosting the plant immune system via the activation of ISR (20). This phyllosphere-originated beneficial effect enhances our understanding of the pattern and mode of colonization by R. palustris strains. As demonstrated in this study, GJ-22 cells underwent a set of morphological and physiological changes to establish a colony in the tobacco phyllosphere.

The phyllosphere epiphytes usually form large heterogeneous aggregates that contain mixed bacterial species. Within the aggregates, multipartite interactions occur among different epiphytic species and the plant, which directly impacts plant health and physiology. It is conceivable that some AHL-producing bacteria that were introduced in the phyllosphere as biocontrol agents may establish AHL-based crosstalk with indigenous microbes to steer the nutritional and microecological conditions toward their own and the host plant’s benefit. In summary, this study revealed that a functioning QS circuit in biocontrol bacteria was required for colonization and pathogen suppression in the phyllosphere. This adds to the current knowledge of the characteristics of QS regulation in the phyllosphere, which mainly addresses the pathogenesis of plant diseases. Furthermore, our findings provoke a broader investigation of the role of the QS circuit in beneficial microorganism-plant interactions, as we believe that the *rpaI-rpaR* QS regulation in R. palustris described in this study is not an exceptional case. The role of bacterial QS regulation should be further investigated for many other biocontrol bacteria and plants.

## MATERIALS AND METHODS

### Bacterial strains, mutant generation, cultural conditions, and inoculum preparation.

R. palustris GJ-22, Escherichia coli DH5α, and E. coli S17-1 were obtained from the Hunan Protection Institute, Hunan Academy of Agricultural Sciences, China. GJ-22 was routinely cultivated aerobically at 30°C on a photosynthetic medium (PM) in a light incubator (PRX-450D, Hangzhou, China) at 6,500 lx. The PM contained the following components (in g L^−1^): 0.1 M (NH_4_)_2_SO_4_, 0.02 M MgSO_4_, 0.5 M Na_2_CO_3_, 0.05 M K_2_HPO_4_, 0.02 M NaCl, 0.2 M Casamino Acids, 0.15 M yeast extract, and 18 g agarose (pH = 6.5 to 7.0). Agar was added to the liquid medium to produce a solid growth medium. When indicated, *p*-coumarate (0.5 mM) or *p*C-HSL (250 nM) was added to the PM. For plasmid propagation, E. coli DH5α cells were aerobically cultivated in Luria-Bertani (LB) broth at 30°C. As donor cells for conjugation, E. coli S17-1 cells were cultivated in LB broth at 37°C. For the GJ-22 mutant cultures, antibiotics were added to the media as indicated.

The bacterial strains and plasmids that were used are listed in Table S1 in Supplementary File 1. The primer pairs that were used for DNA amplification are listed in Table S2 in Supplementary File 1. The plasmid construction, cell conjugation, and electroporation are mentioned in detail in Supplementary File 1.

For the preparation of the bacterial inoculum, GJ-22 or mutant cells were pelleted from the liquid culture at OD_660_ = 0.4 and resuspended in 100 mM phosphate peptone buffer (PPB; pH = 7.2) (Beijing Solarbio Science & Technology Co., Ltd., Beijing, China). For the plant inoculation, the cell density was adjusted to the indicated concentration. For the bacterial liquid culture inoculation, the cell density was adjusted to OD_660_ = 0.4, and the liquid medium was inoculated at a ratio of 5% (vol/vol). When indicated, *p*C-HSL (final concentration: 500 nM) was added to the inoculum. The plants were inoculated by spraying the leaves with the bacterial suspension until they were soaking wet.

### Plant growth.

Seeds of Nicotiana benthamiana were obtained from the Hunan Protection Institute, Hunan Academy of Agricultural Sciences, China. For sterilization, the seeds were placed in a centrifuge tube and shaken in 70% (vol/vol) ethanol/water for 30 min at room temperature. Sterilized seeds were placed on filter paper contained in a petri dish and allowed to air dry for 1 h. Thereafter, the seeds were placed on wet filter paper in a growth chamber at 25°C in the dark and were allowed to germinate for 7 days (humidity: 80 to 90%) (21). For the axenic plant cultivation, each budded seed was transferred to a sterilized pot (10 cm × 10 cm × 15 cm) that contained pH-balanced peat moss (UV irradiation for 30 min) (Klasmann-Deilmann GmbH, Geeste, Germany) inside an aseptic growth chamber that was set at 28°C with a 16/8 h (light/dark) photoperiod and a relative humidity of 85%. For the nonaxenic plant cultivation, budded seeds were cultured normally inside a greenhouse under the same growth conditions as those for the axenic plant cultivation.

### Bacterial sampling, RNA extraction, and RT-PCR for the quantification of target gene expression.

Bacterial cells were sampled by washing the third and fourth leaves of the plants at various hours postinoculation (hpi). 10 grams (fresh weight) of leaves were collected, weighed, and submerged into 200 mL PPB that were contained in a 500 mL conical flask. After 20 min, sonication (47 kHz) was performed for 10 min, and this was followed by shaking at 150 rpm and centrifugation for 10 min at 10,000 × *g*. The leaves were washed three times, and the cells were pooled and resuspended in 2 mL PPB with 20% Percoll. The suspension was centrifuged at 12,000 × *g* for 10 min to separate small plant debris from the bacterial cell pellet.

RNA was extracted from the bacterial cells at the indicated growing stage. A cell pellet was obtained from the 2 mL liquid culture, and cells that were washed from fresh leaves (10 g) were separately suspended in a solution of 2 mL RNAprotect Bacterial Reagent (Qiagen Germany) and 1 mL PPB before total RNA extraction using an RNeasy Mini Kit (Qiagen Germany). Total RNA was treated with 4 units of Turbo DNase (Ambion), and this was followed by quantification using a NanoDrop ND-1000 spectrophotometer (NanoDrop, USA). cDNA was synthesized from 800 ng of total RNA from each sample by using a PrimeScript 1st Strand cDNA Synthesis Kit (TaKaRa Biotechnology [Dalian] Co., Ltd.). The cDNA samples were diluted to 1 ng µL^−1^ and were stored at −20°C.

The transcript levels of the target genes were semiquantified using TransStart Green qPCR SuperMix UDG (TransGen Biotech, Beijing, China) and a PTC-200 real-time PCR system (Bio-Rad, USA). The specific primers and PCR cycling conditions for the target genes and the reference gene (*rpoD*, encoding an RNA polymerase primary sigma factor) are listed in Table S3 in Supplementary File 1. The PCR mixture (20 µL) contained 0.2 µM (each) of the forward and reverse primers and 0.8 µL of the cDNA template. The PCR efficiency for each gene was verified, according to the slope of the standard curve that was generated from the amplification. The transcript levels of the target genes were represented as the fold changes, compared with the reference genes via the 2^−ΔΔCT^ method. The mean deviation was calculated from the standard deviation (SD) in the ΔΔ*CT* value by using the formula 2^−ΔΔCT ±SD^ (22). All of the reactions were performed in triplicate.

### EPS quantification and the determination of the cell density and the bacterial population size.

The total EPS, including the unbound fraction in the culture supernatant and the bound fraction on the cell surfaces, was quantified as described below (21). Bacterial cells were cultured in a 150-mL liquid medium that was contained in a 500-mL conical flask at an inoculation ratio of 5%. EPS was extracted every 12 h for 2 to 9 days postinoculation (dpi). Three flasks of liquid culture (OD_660_ variation of ≤0.01) were used independently at each time point. 1 mL of liquid culture was reserved to determine the cell density, and the rest of the liquid culture was used for EPS extraction. The bacterial cells and the supernatant were separated via centrifugation. The supernatant was mixed with three volumes of absolute ethanol for unbound EPS precipitation. To dislodge the bound EPS from the cell surfaces, the cell pellet was resuspended in 50 mL high-salt buffer (10 mM KPO_4_, 15 mM NaCl, 1 mM MgSO_4_, pH = 7.0) and blended using a Mixer Mill MM400 (Retsch, Haan, Germany) at 0°C for 30 min, using Setting 2. After the removal of the cells from the buffer via centrifugation, the supernatant was mixed with three volumes of ethanol for bound EPS precipitation. Both fractions of EPS were retrieved from viral suspensions via centrifugation at 12,000 × *g* for 30 min, and this was followed by resuspension in 10 mL of sterile water. The EPS were quantified by subjecting the EPS suspension to the phenol-sulfuric acid method (23). In this method, d-glucose was used to generate a standard curve, and the absorbance values of all of the colored viral suspensions were determined using a 96-well microplate spectrophotometer at 488 nm. The cellular protein production of the bacterial cells was determined using a Bradford protein assay. The EPS were quantified as the ratio of EPS to cellular protein.

The bacterial cell density in the liquid culture and the population density on plant leaves were determined by plating and incubating serial dilutions of the cell culture or sampled cell suspension from the leaf surfaces on a PM. The inoculated plates were then incubated at 25°C for 6 d. The CFU were counted, and the cell density and bacterial population size were determined as CFU mL^−1^ and CFU g^−1^ of fresh weight.

### ROI susceptibility test.

The ROI susceptibility of GJ-22 and the mutants was tested as previously described (24). Freshly pelleted bacterial cells from the liquid culture (OD_660_ = 0.4) were resuspended in PPB and were then added to the melted PM agar (0.6% [wt/vol]), thereby resulting in a final concentration of 6 × 10^7^ CFU mL^−1^. 2 mL of this mixture were overlaid on a PM plate. A filter disc (diameter = 0.5 cm) that was soaked with 10% (vol/vol) hydrogen peroxide/water was placed at the center of the plate, and this was followed by incubation. A hydrogen peroxide solution (50 µL) was added to the filter disc every 24 h to maintain the oxidizability. After 4 d of incubation, the diameter of the inhibition zone surrounding the filter disc was measured to evaluate the susceptibility of the bacterial strains to ROIs. All of the photosynthetic media were supplemented with *p*-coumarate and *p*C-HSL when indicated.

### Fluorescence microscopy and confocal laser scanning microscopy (CLSM).

The bacterial cells that were attached to the leaf surface were visualized using a Nikon epifluorescence microscope (Nikon TI-E+C2, Japan) that was coupled to an MRC 1024ES confocal system. Images were obtained using a krypton/argon laser at an excitation wavelength of 488 nm and an emission wavelength of 522/35 nm for eGFP determination.

### Genomic sequencing and analysis.

Genomic DNA was extracted from GJ-22 cells in the liquid culture (OD_660_ = 0.4) by using a TANamp Bacteria DNA Kit (DP302, TIANGEN). The DNA sequencing and the library construction and assembly were performed at Sagene Biotech Co. Ltd. (Guangzhou, China). The low-quality reads were filtered using SMRT Link v5.0.1, and the filtered reads were assembled to generate one contig without gaps (25). Six databases, namely, the Gene Ontology (GO), the Kyoto Encyclopedia of Genes and Genomes (KEGG), the Clusters of Orthologous Groups (COG), the Nonredundant (NR) Protein Database, Pfam, and Swiss-Prot, were used to predict gene function. A whole-genome BLAST search (E value of <1E−6, minimal alignment length percentage of >50%) was performed against the six databases.

The genome information of R. palustris GJ-22 has been deposited into the NCBI under the accession number CP041387. The information regarding the genes is as follows: *Exop1* (NC_018000.1); *Exop2* (NC_020560.1), *rpaI* (NZ_CP066699.1), *ahlI* (NC_005773.3), *ahlR* (NC_005773.3).

### Bioinformatic and statistical analyses.

The sequence alignment and phylogenetic tree were generated by MegAlign (DNASTAR, Inc., Madison, Wisconsin, USA), using the Clustal W method. The alignment result was visualized by BoxShade Server (http://arete.ibb.waw.pl/PL/html/boxshade.html). The known protein sequences were downloaded from NCBI (www.ncbi.nlm.nih.gov/protein).

The data (at least three replicates) were subjected to a one-way analysis of variance (ANOVA), and the pairwise comparison of treatment means was achieved via Fisher’s least significant difference test procedure at a *P* value of < 0.05, using the SPSS Statistics 17.0 software package (IBM Corp., New York, USA). The source data are provided in this paper.

## References

[1] Bodenhausen N, Bortfeld-Miller M, Ackermann M, Vorholt JA. 2014. A synthetic community approach reveals plant genotypes affecting the phyllosphere microbiota. PLoS Genet 10:e1004283. doi:10.1371/journal.pgen.1004283. 24743269 PMC3990490

[2] Lindow SE, Brandl MT. 2003. Microbiology of the phyllosphere. Appl Environ Microbiol 69:1875–1883. doi:10.1128/AEM.69.4.1875-1883.2003. 12676659 PMC154815

[3] Monier JM, Lindow SE. 2004. Frequency, size, and localization of bacterial aggregates on bean leaf surfaces. Appl Environ Microbiol 70:346–355. doi:10.1128/AEM.70.1.346-355.2004. 14711662 PMC321242

[4] Hassani MA, Durán P, Hacquard S. 2018. Microbial interactions within the plant holobiont. Microbiome 6:58. doi:10.1186/s40168-018-0445-0. 29587885 PMC5870681

[5] Bringel F, Couee I. 2015. Pivotal roles of phyllosphere microorganisms at the interface between plant functioning and atmospheric trace gas dynamics. Front Microbiol 6:486. doi:10.3389/fmicb.2015.00486. 26052316 PMC4440916

[6] Müller DB, Vogel C, Bai Y, Vorholt JA. 2016. The plant microbiota: systems-level insights and perspectives. Annu Rev Genet 50:211–234. doi:10.1146/annurev-genet-120215-034952. 27648643

[7] Reisberg EE, Hildebrandt U, Riederer M, Hentschel U. 2013. Distinct phyllosphere bacterial communities on Arabidopsis wax mutant leaves. PLoS One 8:e78613. doi:10.1371/journal.pone.0078613. 24223831 PMC3818481

[8] Remus-Emsermann MN, Tecon R, Kowalchuk GA, Leveau JH. 2012. Variation in local carrying capacity and the individual fate of bacterial colonizers in the phyllosphere. ISME J 6:756–765. doi:10.1038/ismej.2011.209. 22258099 PMC3309366

[9] Lugtenberg B, Kamilova F. 2009. Plant-growth-promoting rhizobacteria. Annu Rev Microbiol 63:541–556. doi:10.1146/annurev.micro.62.081307.162918. 19575558

[10] Vacher C, Hampe A, Porté AJ, Sauer U, Compant S, Morris CE. 2016. The phyllosphere: microbial jungle at the plant–climate interface. Annu Rev Ecol Evol Syst 47:1–24. annurev-ecolsys-121415–032238. doi:10.1146/annurev-ecolsys-121415-032238.

[11] Yi L, Dong X, Grenier D, Wang K, Wang Y. 2021. Research progress of bacterial quorum sensing receptors: classification, structure, function and characteristics. Sci Total Environ 763:143031. doi:10.1016/j.scitotenv.2020.143031. 33129525

[12] Papenfort K, Bassler BL. 2016. Quorum sensing signal-response systems in Gram-negative bacteria. Nat Rev Microbiol 14:576–588. doi:10.1038/nrmicro.2016.89. 27510864 PMC5056591

[13] Hirakawa H, Oda Y, Phattarasukol S, Armour CD, Castle JC, Raymond CK, Lappala CR, Schaefer AL, Harwood CS, Greenberg EP. 2011. Activity of the Rhodopseudomonas palustris p-coumaroyl-homoserine lactone-responsive transcription factor RpaR. J Bacteriol 193:2598–2607. doi:10.1128/JB.01479-10. 21378182 PMC3133176

[14] Schaefer AL, Val DL, Hanzelka BL, Cronan JE, Jr, Greenberg EP. 1996. Generation of cell-to-cell signals in quorum sensing: acyl homoserine lactone synthase activity of a purified Vibrio fischeri LuxI protein. Proc Natl Acad Sci U S A 93:9505–9509. doi:10.1073/pnas.93.18.9505. 8790360 PMC38458

[15] Quiñones B, Dulla G, Lindow SE. 2005. Quorum sensing regulates exopolysaccharide production, motility, and virulence in Pseudomonas syringae. Mol Plant Microbe Interact 18:682–693. doi:10.1094/MPMI-18-0682. 16042014

[16] Larimer FW, Chain P, Hauser L, Lamerdin J, Malfatti S, Do L, Land ML, Pelletier DA, Beatty JT, Lang AS, Tabita FR, Gibson JL, Hanson TE, Bobst C, Torres JL, Peres C, Harrison FH, Gibson J, Harwood CS. 2004. Complete genome sequence of the metabolically versatile photosynthetic bacterium Rhodopseudomonas palustris. Nat Biotechnol 22:55–61. doi:10.1038/nbt923. 14704707

[17] Idi A, Md Nor MH, Abdul Wahab MF, Ibrahim Z. 2015. Photosynthetic bacteria: an eco-friendly and cheap tool for bioremediation. Rev Environ Sci Biotechnol 14:271–285. doi:10.1007/s11157-014-9355-1.

[18] Sasaki K, Watanabe M, Suda Y, Ishizuka A, Noparatnaraporn N. 2005. Applications of photosynthetic bacteria for medical fields. J Bioscience and Bioengineering 100:481–488. doi:10.1263/jbb.100.481. 16384785

[19] Du X, Huang R, Zhang Z, Zhang D, Cheng J, Tian P, Wang Y, Zhai Z, Chen L, Kong X, Liu Y, Su P. 2021. Rhodopseudomonas palustris quorum sensing molecule pC-HSL induces systemic resistance to TMV infection via upregulation of NbSIPK/NbWIPK expressions in Nicotiana benthamiana. Phytopathology 111:500–508. doi:10.1094/PHYTO-05-20-0177-R. 32876530

[20] Su P, Tan X, Li C, Zhang D, Cheng J, Zhang S, Zhou X, Yan Q, Peng J, Zhang Z, Liu Y, Lu X. 2017. Photosynthetic bacterium Rhodopseudomonas palustris GJ-22 induces systemic resistance against viruses. Microb Biotechnol 10:612–624. doi:10.1111/1751-7915.12704. 28296178 PMC5404195

[21] Su P, Zhang D, Zhang Z, Chen A, Hamid MR, Li C, Du J, Cheng J, Tan X, Zhen L, Zhai Z, Tang W, Chen J, Zhou X, Liu Y. 2019. Characterization of Rhodopseudomonas palustris population dynamics on tobacco phyllosphere and induction of plant resistance to Tobacco mosaic virus. Microb Biotechnol 12:1453–1463. doi:10.1111/1751-7915.13486. 31566880 PMC6801143

[22] Livak KJ, Schmittgen TD. 2001. Analysis of relative gene expression data using real-time quantitative PCR and the 2(-delta delta C(T)) method. Methods (San Diego, Calif) 25:402–408. doi:10.1006/meth.2001.1262. 11846609

[23] Jain VM, Karibasappa GN, Dodamani AS, Mali GV. 2017. Estimating the carbohydrate content of various forms of tobacco by phenol-sulfuric acid method. J Educ Health Promot 6:90. doi:10.4103/jehp.jehp_41_17. 29109965 PMC5654475

[24] Qiang Z, Ma L, Celenk M, Chelberg D. 2005. Content-based image retrieval based on roi detection and relevance feedback. Multimed Tools Appl 27:251–281. doi:10.1007/s11042-005-2577-z.

[25] Berlin K, Koren S, Chin CS, Drake JP, Landolin JM, Phillippy AM. 2015. Assembling large genomes with single-molecule sequencing and locality-sensitive hashing. Nat Biotechnol 33:623–630. doi:10.1038/nbt.3238. 26006009

[26] Cai J, Hao Y, Xu R, Zhang Y, Ma Y, Zhang Y, Wang Q. 2022. Differential binding of LuxR in response to temperature gauges switches virulence gene expression in Vibrio alginolyticus. Microbiol Res 263:127114. doi:10.1016/j.micres.2022.127114. 35878491

[27] Ruby EG. 1996. Lessons from a cooperative, bacterial-animal association: the Vibrio fischeri-Euprymna scolopes light organ symbiosis. Annu Rev Microbiol 50:591–624. doi:10.1146/annurev.micro.50.1.591. 8905092

[28] Fuqua WC, Winans SC. 1994. A LuxR-LuxI type regulatory system activates Agrobacterium Ti plasmid conjugal transfer in the presence of a plant tumor metabolite. J Bacteriol 176:2796–2806. doi:10.1128/jb.176.10.2796-2806.1994. 8188582 PMC205432

[29] Zheng H, Zhong Z, Lai X, Chen WX, Li S, Zhu J. 2006. A LuxR/LuxI-type quorum-sensing system in a plant bacterium, Mesorhizobium tianshanense, controls symbiotic nodulation. J Bacteriol 188:1943–1949. doi:10.1128/JB.188.5.1943-1949.2006. 16484206 PMC1426573

[30] González JE, Marketon MM. 2003. Quorum sensing in nitrogen-fixing rhizobia. Microbiol Mol Biol Rev 67:574–592. doi:10.1128/MMBR.67.4.574-592.2003. 14665677 PMC309046

[31] Atkinson S, Chang CY, Sockett RE, Cámara M, Williams P. 2006. Quorum sensing in Yersinia enterocolitica controls swimming and swarming motility. J Bacteriol 188:1451–1461. doi:10.1128/JB.188.4.1451-1461.2006. 16452428 PMC1367215

[32] Williams P, Camara M, Hardman A, Swift S, Milton D, Hope VJ, Winzer K, Middleton B, Pritchard DI, Bycroft BW. 2000. Quorum sensing and the population-dependent control of virulence. Philos Trans R Soc Lond B Biol Sci 355:667–680. doi:10.1098/rstb.2000.0607. 10874739 PMC1692775

[33] Schaefer AL, Greenberg EP, Oliver CM, Oda Y, Huang JJ, Bittan-Banin G, Peres CM, Schmidt S, Juhaszova K, Sufrin JR, Harwood CS. 2008. A new class of homoserine lactone quorum-sensing signals. Nature 454:595–599. doi:10.1038/nature07088. 18563084

[34] Tekel SJ, Smith CL, Lopez B, Mani A, Connot C, Livingstone X, Haynes KA. 2019. Engineered orthogonal quorum sensing systems for synthetic gene regulation in Escherichia coli. Front Bioeng Biotechnol 7:80. doi:10.3389/fbioe.2019.00080. 31058147 PMC6478669

[35] Scott RA, Lindow SE. 2016. Transcriptional control of quorum sensing and associated metabolic interactions in Pseudomonas syringae strain B728a. Mol Microbiol 99:1080–1098. doi:10.1111/mmi.13289. 26713670

