# Supplementary material for: Quorum Sensing Is Required for the Colony Establishment of a Plant Phyllosphere Bacterium Rhodopseudomonas palustris Strain GJ-22
Source: Appl Environ Microbiol. 2023 Jun 5;89(6):e00487-23. doi: 10.1128/aem.00487-23 (PMC10305029; doi:10.1128/aem.00487-23)
Supplement: Supplemental file 1 — Supplemental material. Download aem.00487-23-s0001.pdf, PDF file, 0.4 MB [file aem.00487-23-s0001.pdf]

Supplementary Material

Supplementary file 1

Mutant generation

Plasmids pSUP202I, pSUP202E1, pSUP202E2, pSUP202R, and pSUP202couB construction

Two DNA fragments, 343 bp upstream flanking region and 365 bp downstream flanking region of rpaI gene CDS were PCR-amplified from GJ-22 genome (GeneBank Accession number: CP041387) with specially designed primer pairs. The Hyg^r gene following Lac promoter was PCR-amplified from pCAMBIA1300. The plasmid pSUP202 was linearized with BamH I and Sal I. The four obtained fragments were assembled with Trelief ^{TM} SoSoo Clining Kit (Beijing TsingKe Biotech Co., Ltd., China) by following kit instruction. Fifty nanogram of linearized pSUP202 was used in this reaction. The molar ratio of linearized plasmid and the other three DNA fragments was 5:1:1:1. The reaction system was 5 μL 2 × SoSoo Mix mixed with the four DNA fragment solutions, and adding dd H_2O up to 10 μL. The reaction condition was 50 °C for 45 min. The assembled plasmid pSUP202I was then introduced into competent S17-1 cells.

The plasmids pSUP202E1, pSUP202E2, pSUP202R, and pSUP202couB were constructed with the same method described above. Except, the upstream flanking region and downstream flanking region were 320 bp and 324 bp for *rpaR* CDS; 366 bp and 271 bp for *couB* CDS; 202 bp and 289 bp for *Exop1* CDS; 253 bp and 212 bp for *Exop2* CDS. The antibiotic resistant gene cassette was Amp^r gene following pAMP promoter for replacing *rpaR* gene CDS and *couB* CDS and Hyg^r gene following Lac promoter for replacing *Exop1* CDS and *Exop2* CDS.

Plasmid pBBR1MCS-2I, pBBR1MCS-2E1, pBBR1MCS-2E2, pBBR1MCS-2R, pBBR1MCS-2couB construction

The genes were PCR-amplified from GJ-22 genome with specially designed primer pairs. The plasmid pBBR1MCS-2 was linearized with Hind III and EcoR I. The amplified gene fragment and the linearized pBBR1MCS-2 were assembled with Trelief ^{TM} SoSoo Clining Kit as described for Plasmid pSUP202I construction. The molar ratio of linearized plasmid and the DNA fragments was 5:1. The assembled plasmid pBBR1MCS-2I and pBBR1MCS-2R were then introduced into electro-competent DH5α cells for propagation.

Cell conjugation and transformation

The conjugation of GJ-22 and S17-1 containing suicide plasmids was conducted through the following procedure. GJ-22 and S17-1 cells were pelleted from 50 mL liquid culture during logarithmic growth by centrifugation. Both cell pellets were suspended in 5 mL PPB individually and mixed at the ratio of GJ-22:S17-1 = 3:1. The mixture was filtered onto a nitrocellulose disc (0.22 μm pore size). The disc was then incubated mating side up on PM under 30 °C, 6,500 lux. After 10 h incubation, suspended with 2 mL PPB. The suspension was spread on PM plate supplemented with antibiotics and cultivated under under 30 °C, 6,500 lux to select the recombinants. The type and concentration of antibiotics were 100 μg mL-1 hygromycin for ΔrpaI and 100 μg mL-1 ampicillin for ΔrpaR. The type and concentration of antibiotics were 100 μg mL^{-1} hygromycin for Δ*rpaI* and 100 μg mL^{-1} ampicillin for Δ*rpaR.*

The transformation of GJ-22 wild type and its mutants with plasmid pBBR1MCS-2I, pBBR1MCS-2E1, pBBR1MCS-2E2, pBBR1MCS-2R, pBBR1MCS-2couB, pBBR1MCS-2-pAMP-EGFP was conducted through the following procedure. Cells were pelleted from liquid culture at OD_{660} = 0.4, then rinsed three times in ice-cold sterile water before resuspended in 10% (v/v) glycerol solution and cells in the suspension was electro-competent and immediately stored at -80 °C for later use; 50 µL electro-competent cell suspension was thaw on ice for 20 mins and mixed with 5 ng plasmid DNA in a 2 mm Gene Pulser cuvette containing 80 µL ice-cold sterile water; after placing the cuvette on ice for 10 mins for incubation, cell electroporation was completed by subjecting the cuvette to Eppendorf Eporator (Eppendorf North America, Hauppauge, NY, USA) at 2.5 KV voltage, 25 µF capacitance, and 100 Ω resistance; cell suspension were added into 10 mL liquid medium and cultured for 20 h at 30 °C with 6,500 lux in light incubator. The positive transformants were finally selected on agar medium with 50 $\mu g \cdot mL^{-1}$ kanamycin under the same culture condition. The stability of mutants was analyzed by cultivating in liquid medium with and without kanamycin for 20 generations. For EGFP labeled strains, fluorescence of each generation was examined with laser scanning confocal microscope (LSCM) using excitation at 488 nm-emission 522/35 nm for EGFP (Nikon C2 plus, Nikon, Japan),

In Table S1, bacterial strains and plasmids involved in this research were listed. In Table S2, primer pairs used for DNA fragment amplification were listed. Table S3. Primers used for RT-PCR and PCR cycling condition.

Table S1. Bacterial strains and plasmids

**Bacterial strains and Plasmids**	**Relevant characteristics**[a]	**Reference or source**
Rhodopseudomonas palustris		
**GJ-22（CP041387)**	Wilde type (WT)	This study
**Δ*rpaI***	Δ*rpaI*::Hyg^r, derivative of GJ-22, Hyg^r gene controlled by CaMV 35S promoter	This study
**Δ*rpaR***	Δ*rpaR*::Amp^r, derivative of GJ-22, Amp^r gene controlled by pAMP promoter	This study
**Δ*Exop1***	Δ*Exop1*:: Hyg^r, derivative of GJ-22, Hyg^r gene controlled by CaMV 35S promoter	This study
**Δ*Exop2***	Δ*Exop**2***:: Hyg^r, derivative of GJ-22, Hyg^r gene controlled by CaMV 35S promoter	This study
**Δ*couB***	Δ*couB*:: Amp^r, derivative of GJ-22, Amp^r gene controlled by pAMP promoter	This study
**WT-EGFP**	km^r, GJ-22 harboring pBBR1MCS-2-pAMP-EGFP	This study

**Δ*rpaI*-EGFP**	km^r, Hyg^r, Δ*rpaI* harboring pBBR1MCS-2-pAMP-EGFP	This study
**Δ*Exop1* - EGFP**	km^r, Hyg^r, Δ*Exop1* harboring pBBR1MCS-2-pAMP-EGFP	This study
Escherichia coli		
**DH5α**	Plasmid propagation	ThermoFisher Scientific, USA
**S17-1**	Transformation host, conjugation	ATCC47055
**Plasmids**		
**pClone007**	Amp^r, cloning vehicle	Beijing TsingKe Biotech Co., Ltd.
**pMD18T**	Amp^r, Source of Amp^r cassette	Beijing TsingKe Biotech Co., Ltd.
**pCAMBIA1300**	Km ^{r}, Source of Hyg^r cassette	Beijing TsingKe Biotech Co., Ltd.
**pSUP202**	Amp^r, suicide plasmid	Beijing TsingKe Biotech Co., Ltd.
**pSUP202I**	Amp^r, Hyg^r, in-frame Δ*rpaI*::Hyg^r in pSUP202, CaMV 35S promoter	This study
**pSUP202R**	Amp^r, in-frame Δ*rpaR*::Hyg^r in pSUP202, pAMP promoter	This study
**pSUP202E1**	Amp^r, Hyg^r, in-frame Δ*Exop1*::Hyg^r in pSUP202, CaMV 35S promoter	This study
**pSUP202E2**	Amp^r, Hyg^r, in-frame Δ*Exop2*::Hyg^r in pSUP202, CaMV 35S promoter	This study
**pSUP202couB**	Amp^r, in-frame Δ*couB*::Hyg^r in pSUP202, pAMP promoter	This study
**pBBR1MCS-2**	Km ^{r}, broad-host range expression vector	Beijing TsingKe Biotech Co., Ltd.
**pBBR1MCS-2I**	Km ^{r}, pBBR1MCS-2 derivative, complementation for Δ*rpaI*::Hyg^r, harboring rpaI gene and 86 bp upstream of rpaI gene CDS controlled by lac promoter	This study
**pBBR1MCS-2R**	Km ^{r}, pBBR1MCS-2 derivative; complementation for Δ*rpaR*::Amp^r, harboring rpaR gene controlled by lac promoter	This study

**pBBR1MCS-2E1**	Km ^{r}, pBBR1MCS-2 derivative, complementation for Δ*Exop1*::Hyg^r, harboring Exop1 gene controlled by lac promoter	This study
**pBBR1MCS-2E2**	Km ^{r}, pBBR1MCS-2 derivative, complementation for Δ*Exop2*::Hyg^r, harboring Exop2 gene controlled by lac promoter	This study
**pBBR1MCS-2C**	Km ^{r}, pBBR1MCS-2 derivative; complementation for Δ*couB*::Amp^r, harboring couB gene controlled by lac promoter	This study
**pBBR1MCS-2-pAMP-EGFP**	Km ^{r}, pBBR1MCS-2 derivative, harboring egfp gene controlled by pAMP promoter	Beijing TsingKe Biotech Co., Ltd.

[a] km^r, kanamycin resistant; Amp^r, ampicillin resistant; Hyg^r, hygromycin resistant. (The previously described photosynthetic bacterium *R. palustris* strain GJ-22 was from Hunan Protection Institute, Hunan Academy of Agricultural Sciences, China)

Table S2. Primers used for plasmid construction

**List**	**Sequence (5’ to 3’)**	**Note [a]**
**For pSUP202I construction**		
**UP-rpaI-F**	5’-CGACCACACCCGTCCTGTGGATCC GAGGGCTATTGCATTCCGCT-3’	Forward primer for upstream flanking region; the red labeled fragment overlaps sequence from the BamH I digested end on pSUP202; sequence in small letters is BamH I restriction site
**UP-rpaI-R**	5’-TATTACCCTTTGTTGAAAAGTCTCA ATTGCAGGATCTCGCATTCC-3’	Reverse primer for upstream flanking region; the red labeled fragment reversely complements the first 25 bp of CaMV 35S promoter of Hyg^r gene
**DOWN-rpaI-F**	5’-CGTCCGAGGGCAAAGAAATAG ACGCTTCGTCCAAGCCCC-3’	Forward primer for downstream flanking region; the red labeled fragment overlaps 21 bp of the end of Hyg^r gene
**DOWN-rpaI-R**	5’-GGCTCTCAAGGGCATCGGTCGAC GATGCCCTCAAAAGCCACG-3’	Reverse primer for downstream flanking region; the red labeled fragment reversely complements sequence from the Sal I digested end on pSUP202; sequence in small letters is Sal I restriction site
**For pSUP202E1 construction**		
**UP-Exop1-F**	5’-CGACCACACCCGTCCTGTGGATCC ACTGCAACGTGTTCCCGGAG-3’	Forward primer for upstream flanking region; the red labeled fragment overlaps sequence from the BamH I digested end on pSUP202; sequence in small letters is BamH I restriction site
**UP- Exop1-R**	5’-TATTACCCTTTGTTGAAAAGTCTCA TGCATCGCTCCACAAGGCC-3’	

**DOWN-Exop1-F**	5’-CGTCCGAGGGCAAAGAAATAG CCGATAGGACCGCTCCGATT-3’	Reverse primer for upstream flanking region; the red labeled fragment reversely complements the first 25 bp of CaMV 35S promoter of Hyg^r gene
**DOWN-Exop1-R**	5’-GGCTCTCAAGGGCATCGGTCGAC GGAATGATCTGGATGGCCGC-3’	Forward primer for downstream flanking region; the red labeled fragment overlaps 21 bp of the end of Hyg^r gene
		Reverse primer for downstream flanking region; the red labeled fragment reversely complements sequence from the Sal I digested end on pSUP202; sequence in small letters is Sal I restriction site
**For pSUP202E2 construction**		
**UP-Exop1-F**	5’-CGACCACACCCGTCCTGTGGATCC ATATCTCGCCAAGAATCCCG-3’	Forward primer for upstream flanking region; the red labeled fragment overlaps sequence from the BamH I digested end on pSUP202; sequence in small letters is BamH I restriction site
**UP- Exop1-R**	5’-TATTACCCTTTGTTGAAAAGTCTCA TGGAGCTATCCCTCGCCAC-3’	Reverse primer for upstream flanking region; the red labeled fragment reversely complements the first 25 bp of CaMV 35S promoter of Hyg^r gene
**DOWN-Exop1-F**	5’-CGTCCGAGGGCAAAGAAATAG CAGCCCGCCCCTCCAAAAAC-3’	Forward primer for downstream flanking region; the red labeled fragment overlaps 21 bp of the end of Hyg^r gene
**DOWN-Exop1-R**	5’-GGCTCTCAAGGGCATCGGTCGAC GCTGGTCTATACCGGTGCCC-3’	Reverse primer for downstream flanking region; the red labeled fragment reversely complements sequence from the Sal I digested end on pSUP202; sequence in small letters is Sal I restriction site
**Hyg-F**	5’-TGAGACTTTTCAACAAAGGGTAATA-3’	Forward primer for Hyg^r gene cassette with CaMV 35S promoter
**Hyg-R**	5’-CTATTTCTTTGCCCTCGGACG-3’	Reverse primer for Hyg^r gene cassette with CaMV 35S promoter
**For pSUP202R construction**		
**UP-rpaR-F**	5’-CGACCACACCCGTCCTGTGGATCC ATCGATGTAATCGACGGCTAC-3’	Forward primer for upstream flanking region; the red labeled fragment overlaps sequence from the BamH I digested end on pSUP202; sequence in small letters is BamH I restriction site
**UP-rpaR-R**	5’-AAACAAATAGGGGTTCCGCG AACCTCCGATCCGATAACTGC-3’	Reverse primer for upstream flanking region; the red labeled fragment reversely complements the first 20bp of pAMP promoter of Amp^r gene
**DOWN-rpaR-F**	5’-GCCTCACTGATTAAGCATTGGTAA CCTGTCCGATCGGACAGTAGTT-3’	Forward primer for downstream flanking region; the red labeled fragment overlaps 24 bp of the end of Amp^r gene
**DOWN-rpaR-R**	5’-GGCTCTCAAGGGCATCGGTCGAC AGCAGCGTGGGCGAGGTG-3’	Reverse primer for downstream flanking region; the red labeled fragment reversely complements sequence from the Sal I digested end on pSUP202; sequence in small letters is Sal I restriction site

**For pSUP202couB construction**		
**UP-rpaR-F**	5'-CGACCACACCCGTCCTGTGGATCC GCTGCCGTGCTGCACGAT-3'	Forward primer for Amp^r gene cassette with pAMP promoter
**UP-rpaR-R**	5'-AAACAAATAGGGGTTCCGCG TTCGGTCTCCACGTTTCTTG-3'	Reverse primer for Amp^r gene cassette with pAMP promoter
**DOWN-rpaR-F**	5'-GCCTCACTGATTAAGCATTGGTAA CTCCTCGGCTCAACGTCATT-3'	
**DOWN-rpaR-R**	5'-GGCTCTCAAGGGCATCGGTCGAC GCCCAGTGGAAGCAGATCG-3'	
**Amp-F**	5'-CGCGGAACCCCTATTTGTTT-3'	Forward primer for Amp^r gene cassette with pAMP promoter
**Amp-R**	5'-TTACCAATGCTTAATCAGTGAGGC-3'	Reverse primer for Amp^r gene cassette with pAMP promoter
**For pBBR1MCS-2I construction**		
**rpaI-F**	5'-TCGAGGTCGACGGTATCGATAAGCTT ACCTGTCCGATCGGACAGTAGT-3'	Forward primer for 86 bp upstream sequence and rpaI gene amplification; the red labeled fragment overlaps sequence of the Hind III digested end on pBBR1MCS-2; sequence in small letter is Hind III restriction site
**rpaI-R**	5'-CCCCCGGGCTGCAGGAATTC TCATCGGATCACCTGAAATTCC-3'	Reverse primer for rpaI gene amplification; the red labeled fragment reversely complements the EcoR I digested end on pBBR1MCS-2; sequence in small letters is EcoR I restriction site
**For pBBR1MCS-2E1 construction**		
**Exop1-F**	5'-TCGAGGTCGACGGTATCGATAAGCTT ATGAGGTTGCCCGTGGTG-3'	Forward primer for 86 bp upstream sequence and rpaI gene amplification; the red labeled fragment overlaps sequence of the Hind III digested end on pBBR1MCS-2; sequence in small letter is Hind III restriction site
**Exop1-R**	5'-CCCCCGGGCTGCAGGAATTC TCACCAACGGGTCTTCGTGG-3'	Reverse primer for rpaI gene amplification; the red labeled fragment reversely complements the EcoR I digested end on pBBR1MCS-2; sequence in small letters is EcoR I restriction site
**For pBBR1MCS-2E2 construction**		
**Exop2-F**	5'-TCGAGGTCGACGGTATCGATAAGCTT ATGCGTGTCGTAGGTGCGTT-3'	Forward primer for 86 bp upstream sequence and rpaI gene amplification; the red labeled fragment overlaps sequence of the Hind III digested end on pBBR1MCS-2; sequence in small letter is Hind III restriction site

**Exop2-R**	5’-CCCCCGGGCTGCAGGAATTC TTAGAACCAGCGTTCACCGA-3’	Reverse primer for rpaI gene amplification; the red labeled fragment reversely complements the EcoR I digested end on pBBR1MCS-2; sequence in small letters is EcoR I restriction site
**For pBBR1MCS-2R construction**		
**rpaR-F**	5’-TCGAGGTCGACGGTATCGATAAGCTT ATGATCGTCGGCGAGGATC-3’	Forward primer for 86 bp upstream sequence and rpaI gene amplification; the red labeled fragment overlaps sequence of the Hind III digested end on pBBR1MCS-2; sequence in small letter is Hind III restriction site
**rpaR-R**	5’-CCCCCGGGCTGCAGGAATTC TCACAGCCGGATCAATCCAA-3’	Reverse primer for rpaI gene amplification; the red labeled fragment reversely complements the EcoR I digested end on pBBR1MCS-2; sequence in small letters is EcoR I restriction site
**For pBBR1MCS-2couB construction**		
**couB-F**	5’-TCGAGGTCGACGGTATCGATAAGCTT ATGCCCGACTTCATCACTCTTC-3’	Forward primer for 86 bp upstream sequence and rpaI gene amplification; the red labeled fragment overlaps sequence of the Hind III digested end on pBBR1MCS-2; sequence in small letter is Hind III restriction site
**couB-R**	5’-CCCCCGGGCTGCAGGAATTC TTACGACGCCGCCTTCAT-3’	Reverse primer for rpaI gene amplification; the red labeled fragment reversely complements the EcoR I digested end on pBBR1MCS-2; sequence in small letters is EcoR I restriction site

[a] km^r, kanamycin resistant; Amp^r, ampicillin resistant; Hyg^r, hygromycin resistant.

Table S3. Primers used for RT-PCR and PCR cycling condition

List	Sequence (5’ to 3’)	PCR cycling condition
***rpaI* (189 bp)**		
**Forward**	5’-CGAGGGCCAGTTGAGGAAAT-3’	Initial polymerase activation step at 95 °C for 1 min followed by 40 cycles of 95 °C for 10 s, 60 °C for 15 s; fluorescence signal detection at 60°C.
**Reverse**	5’-AGATCTACGTCGTCGAACGC-3’	
***Exop1*(156 bp)**		
**Forward**	5’-GAGACATCGACCAGCGCATA-3’	Initial polymerase activation step at 95 °C for 1 min followed by 40 cycles of 95 °C for 10 s, 60 °C for 15 s; fluorescence signal detection at 60°C.
**Reverse**	5’-GACGTTTCAGGTGCTTGCTG-3’	

***Exop2* (159 bp)**		
**Forward**	5’-ACACCGAACACCCTCGATTC-3’	Initial polymerase activation step at 95 °C for 1 min followed by 40 cycles of 95 °C for 10 s, 60 °C for 15 s; fluorescence signal detection at 60°C.
**Reverse**	5’-ATTGGTGAGTCCTTCCTGGC-3’	
**Reference gene *rpoD* (122 bp)**		
**Forward**	5’-GGTCTCGAGATCGGCGAATT-3’	Initial polymerase activation step at 95 °C for 1 min followed by 40 cycles of 95 °C for 10 s, 60 °C for 15 s; fluorescence signal detection at 60°C.
**Reverse**	5’-TTGGCGATCGAGATCACGAG-3’	

Supplementary file 2

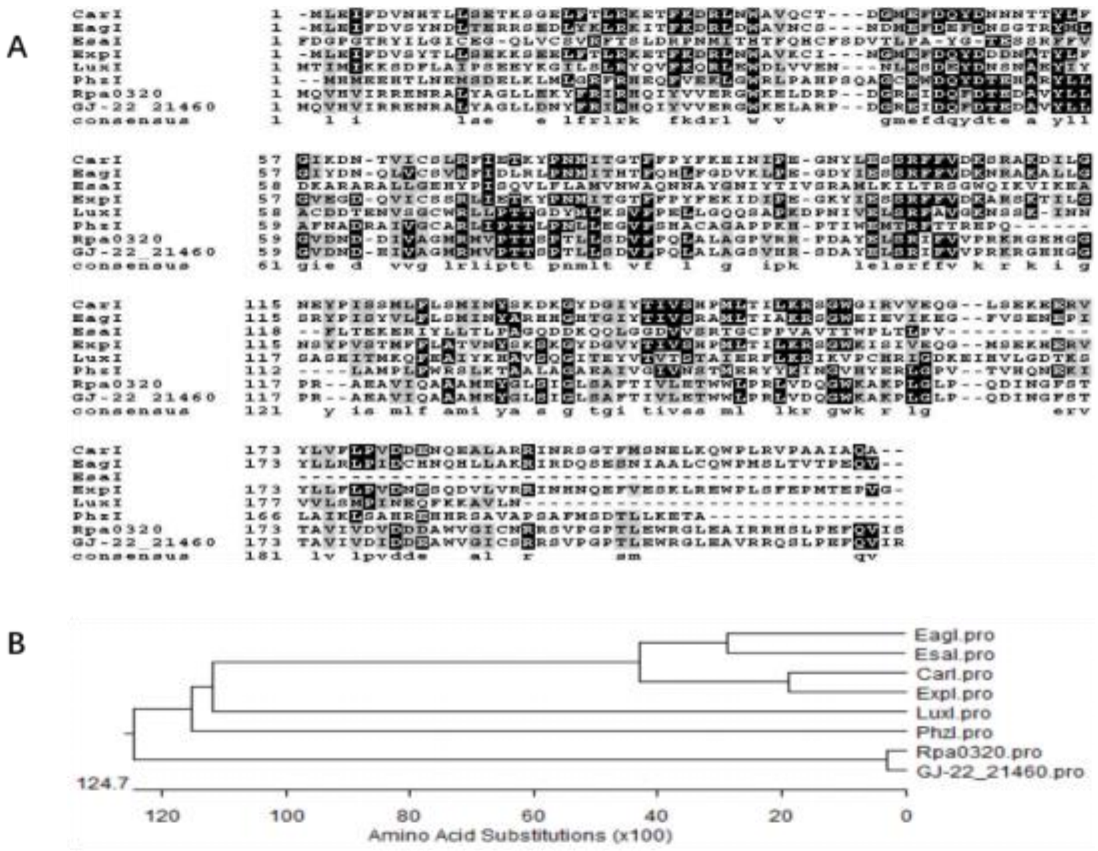

Figure S1. The Amino acid sequence alignment and phylogenetic tree of LuxI family proteins. (A) CarI from *Erwinia carotovora*, protein id= CAA52352.1; EagI from *Pantoea agglomerans*, protein id= CAA52353.1; EsaI from *Pantoea stewartia*, protein id= AAA82096.1; ExpI from *Erwinia carotovora*, protein id= CAA51409.1; LuxI from *Vibrio fischeri*, protein id= AAP22376.1; PhzI from *Pseudomonas sp.*, protein id= ACI88827.1; YenI from *Yersinia enterocolitica*, protein id= CAA53695.1; Rpa0320 represents rpaR from *Rhodopseudomonas palustris* CGA009, protein id= CAE25764.1; GJ-22_21460 represents the luxI homologue from *R. palustris* strain GJ-22. (B) phylogenetic tree of aligned luxI homologues. The known protein sequences were downloaded from NCBI (www.ncbi.nlm.nih.gov/protein) with protein id; sequence alignment and phylogenetic tree were generated with MegAlign by Clustal W method, and the shadow box was generated with BoxShade Sever (https://embnet.vital-it.ch/software/BOX_form.html).

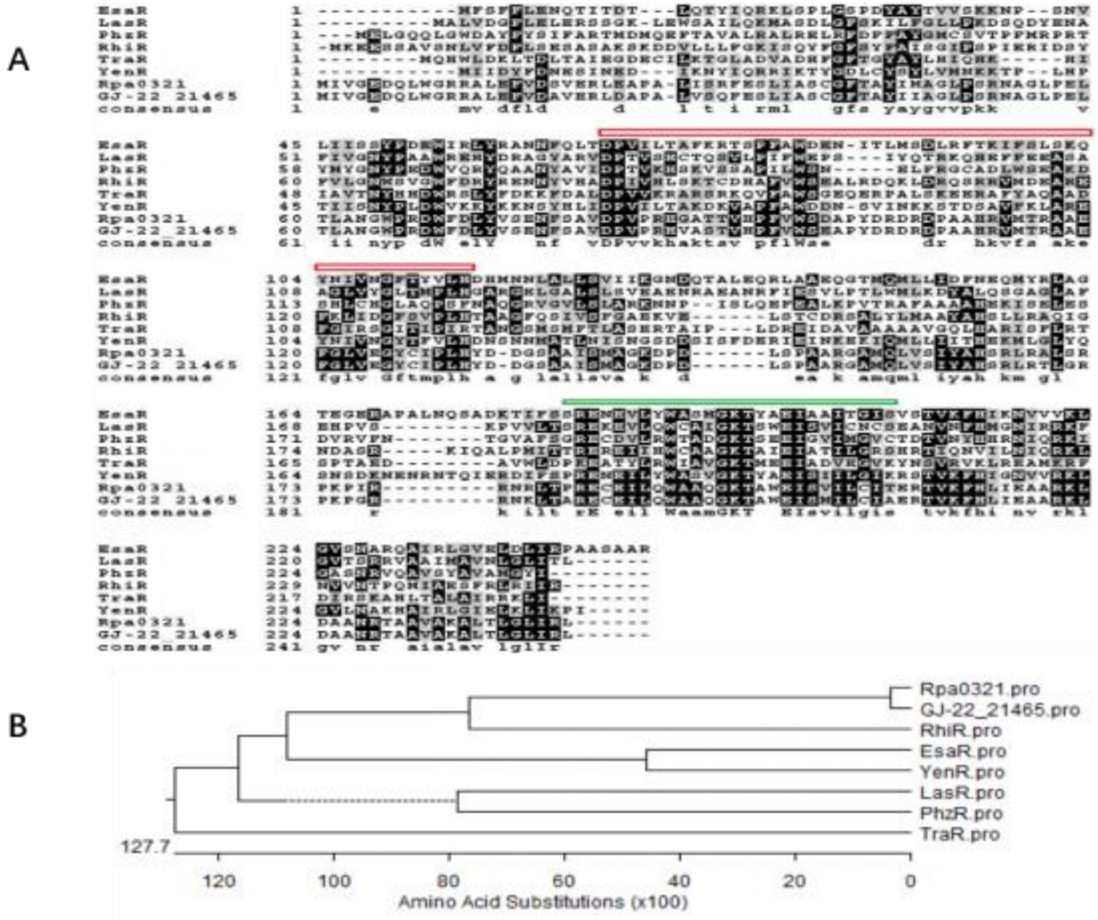

Figure S2. The Amino acid sequence alignment and phylogenetic tree of-proteins. (A) The secondary structure of the target protein was revealed by ClustalX2 and ESPpript 3.0(39). EsaR from *Pantoea stewartia*, protein id= AAA82097.1; LasR from *Pseudomonas aeruginosa*, protein id= BAA06489.1; PhzR from *Pseudomonas chlororaphis*, protein id= AEX57289.1; RhiR from *Rhizobium leguminosarum* bv. Viciae, protein id= AAA26360.2; TraR from *Agrobacterium radiobacter*, protein id= AAD31600.1; YenR from *Yersinia enterocolitica,* protein id= CAA53694.1; Rpa0321 represents rpaR from *Rhodopseudomonas palustris* CGA009, protein id= CAE25765.1; GJ-22_21465 represents the luxR homologue from *R. palustris* strain GJ-22; AA residues marked with red bar are the autoinducer-binding domain for AHLs, residues marked with green bar are the helix-turn-helix (HTH) DNA binding motifs at the C termimal of LuxR family regulator. (B) phylogenetic tree of aligned luxI homologues. The known protein sequences were downloaded from NCBI (www.ncbi.nlm.nih.gov/protein) with protein id; sequence alignment and phylogenetic tree were generated with MegAlign by Clustal W method, and the shadow box was generated with BoxShade Sever (https://embnet.vital-it.ch/software/BOX_form.html).

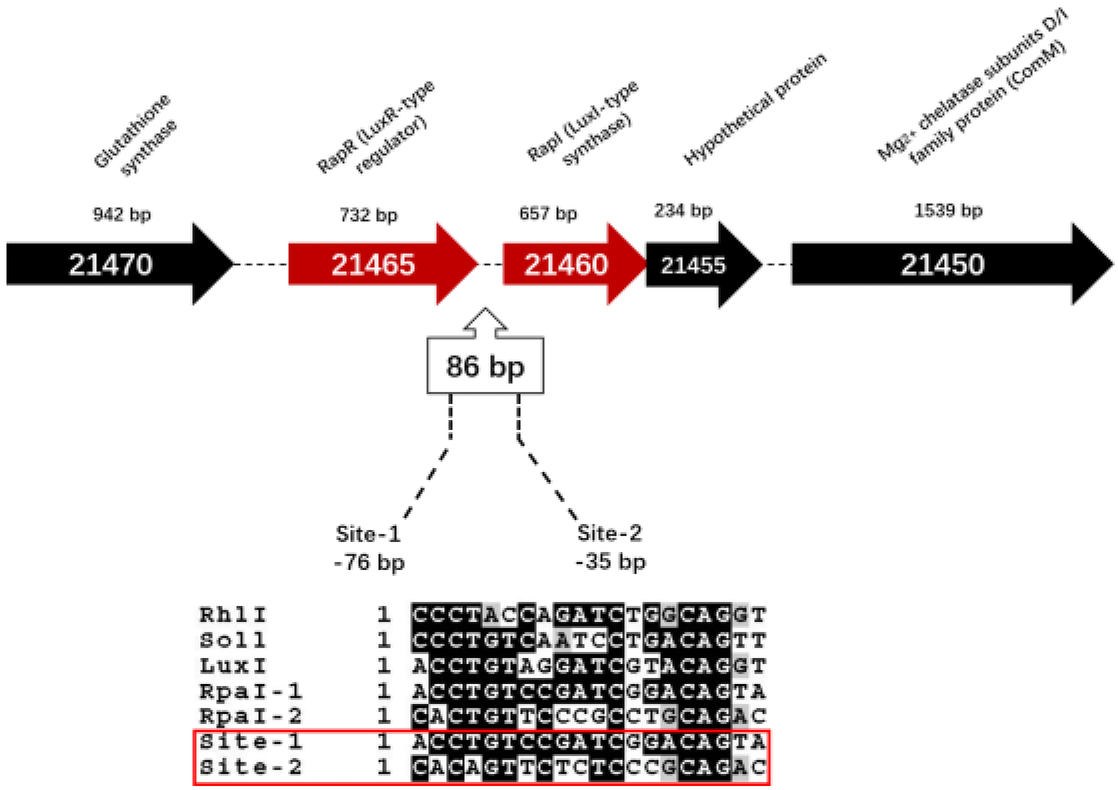

Figure S3. The *rpaR-lux box-rpaI* gene arrangement in *Rhodopseudomonas palustris* strain GJ-22 genome.The red arrow bars represent *rpaR* and *rpaI* homologues in *R. palustris* GJ-22 genome with their locus tag: GJ-22_21465 and GJ-22_21460; the back arrow bars represent genes flanking the *rapR-rpaI* gene pairs, numbers in bars represent their locus tag: GJ-22_21470, GJ-22_21455, GJ-22_21450; the 86-bp sequence sits between *rpaR* and *rpaI* contains two lux-box like elements centering at – 76 and – 35 bp upstream of *rpaI* ATG start codon; the aligned sequences are known lux-box like elements from *Pseudomonas aeruginosa* (RhlI), *Ralstonia solanacearum* (SolI), *Vibrio fischeri* (LuxI), *Rhodopseudomonas palustris* CGA009 (RpaI-1 and RpaI-2), sequences shown in red square are two lux-box like elements in *R. palustris* GJ-22; sequence alignment were generated with MegAlign by Clustal W method, and the shadow box was generated with BoxShade Sever (https://embnet.vital-it.ch/software/BOX_form.html).
